# The Institutional Library

**Published:** 1914-03-07

**Authors:** 


### THE INSTITUTIONAL LIBRARY.

### The Hygiene of Schools.

School Hygiene. By Fletcher B. Dresslar, Ph.D. (New York: The Macmillan Co. 1913. Illustrated. Pp. 368. Price 5s. 6d.)

It is not very flattering to British experts in school hygiene and school sanitation to find that it is left for an American to produce the first book which deals with these subjects in the way in which they should be treated. It is not suggested that all the details and the opinions to be found in Dr. Dresslar's book are to be held up as an example to English hygienists, for the book naturally deals with American conditions, and in the United States school matters are in a much more "advanced" stage than they are with us. But this text-book is a "live" volume; it appeals directly to the intelligence and common sense of the teachers and others for whom it has been written. One feels that the author really has made a study of school hygiene. This manual is not one of those compilations, of which there exist not a few in this country, containing a bit of physics, a bit of sanitation, and some elementary anatomy and physiology. A distinctive feature is to be found at the close of each chapter in the shape of a list of suggestions arising out of the foregoing subject-matter and affording stimulating topics for further investigation. One of the completest bibliographies we have seen in connection with school subjects is here distributed in sections at the end of each chapter throughout the book, so that a teacher who wishes to investigate special prints or to study comparative methods need not waste any time in searching the literature. This alone adds very greatly to its practical value.

The author admits that he has not attempted to deal with any phase of the subject exhaustively, but we must congratulate him on the thoroughly practical attention he has given to many miscalled minor details, which are, nevertheless, of the greatest moment to the health of the children. For example, the dissertation on blackboards, chalk, and dusters shows an appreciation of the importance of these matters; there is no mention, however, of the value or otherwise of mixing the chalk with silicate, which by loading the dust makes it fall more quickly. On damp days a chalk fog is readily caused by condensation of water on chalk dust. In the construction of school buildings the uses of a basement are intelligently insisted upon, and another small point which is too often neglected receives mention—namely, the need of gutters below the eaves and means for preventing rain from soaking into the ground near the walls—one would have thought this was an obvious proposition, but a visit to a few schools in this country will prove that this detail cannot be taken for granted. The subject of school desks has been written upon *ad nauseam*, and the chapter dealing with this gives the author's personal views both from a scientific and practical standpoint. He favours the sloping desk. Although this chapter is an excellent one, it must be admitted that the best discussion on the subject of measurements for school desks is to be found in Ragazzi's "L'Igiene della scuola e dello scolaro."

On the hygiene of instruction, on open-air schools, and on the importance of organised games Dr. Dresslar is most interesting, but a good deal of the matter applies solely to American children. It is somewhat curious to find that the school nurse is barely touched upon, although there is a chapter on medical inspection. Medical subjects are fortunately only lightly dealt with; if teachers wish to become amateur physicians they will find plenty of material elsewhere. There is, however, a chapter devoted to the care of the teeth and a photograph showing teachers being taught tooth-brush drill. This book will, we doubt not, take a foremost place among the literature of school hygiene, and will remain for a long period an authoritative work on school construction and the hygiene of scientific pedagogy.

Noted Murder Mysteries. By Philip Curtin. (London: Simpkin, Marshall and Co. 7s. 6d.)

Medical and scientific testimony play an ever increasing part in the disentanglement of the mysteries of crime, and any book which records a series of criminal trials, selected with particular reference to the "poignant illustrations of human nature" which they convey, must of necessity attract the interest of many medical readers. But it is not so much with the motive for the crime that the scientific witness is concerned as with establishing or rebutting theories as to the cause of death, which can only be tested satisfactorily, in cases of suspected poisoning, at any rate, with the aid of chemical and biological testimony. To this extent, therefore, this book does not possess any exclusive value to members of the medical profession. None the less, the way in which the various cases are treated will make the book appeal to students of criminology, and the fact that the author has selected nine separate trials of such widely different kinds as the celebrated Lafarge case, the little-known mystery connected with the death of Mr. Charles Bravo, and a typical instance of the *crime passionnel*, serves to minimise the morbidity which might otherwise be expected to predominate in a work of this nature.

### The Pocket Guide Series.

"The Nursing Mirror" Pocket Encyclopædia and Diary, 1914.

The Midwife's Pocket Encyclopædia and Diary, 1914. (The Scientific Press. Price 6d. each net.)

The former of these two handy and useful little diaries is now an established favourite in its seventh year of publication. It has been carefully revised, is thoroughly trustworthy, and is a veritable mine of information on subjects connected with nursing. The latter is a newcomer this year, and has made a most auspicious start. The arrangement of the diary part is just the same as in the former case. The compendium of information which precedes the actual diary is wonderfully compact and complete. We unhesitatingly predict for this publication a popularity as great as that which its companion booklet has already attained.

Anatomy, Descriptive and Applied. By H. Gray, F.R.C.S. Eighteenth Edition. Edited by R. Howden, M.A., D.Sc., C.M. Notes on Applied Anatomy revised by Dr. A. J. Jex-Blake, F.R.C.P., and W. F. Fedden, M.S., F.R.C.S. (London: Longmans, Green and Co. 1913. Price 32s. net.)

Yet another Gray! How many hundreds of medical men, now established in practice, have been brought up on "Gray's Anatomy," which "they hated so, not for its faults but theirs"! And still the editions go on. Fortunately it is so, for as a text-book it is still unbeaten, if not now unrivalled. It is interesting to watch its progress. The present edition is nearly double the size of the first, and contains treble the number of illustrations, of which over four hundred are coloured. To old friends of this work perhaps the greatest change will be the introduction of what is known as the "Basle"

nomenclature. This nomenclature has already been adopted by several other anatomical works, and has evidently come to be accepted and must be learnt by modern students. No doubt future generations will see further modifications, but at present it forms a useful step towards international standardisation and makes for lucidity. Another change, which may or may not be considered an advantage, is the collection of all the paragraphs relating to the surface anatomy of the various organs into one special chapter. This section, always of the greatest importance to students, has been well revised by Dr. Jex-Blake and Mr. W. F. Fedden. There need be no apprehension on the part of students that "Gray's Anatomy" has been improved beyond recognition, nor that it will fail to justify its purchase.

### Two Manuals on Cleanliness.

British Red Cross Society, Manual IV.; Hygiene and Sanitation. By Lieutenant-Colonel S. G. Moores, R.A.M.C. (London: Cassell and Co. Price 1s. net.)

The previous Red Cross Society manuals, in their attractive light-blue linen covers, have naturally led up to one on hygiene and sanitation; and the Voluntary Aid Detachments must have profited greatly by their text-books on the various subjects which they are expected to study. This latest member of the series is an excellent shilling's worth. It is concise, clear, and practical; quite evidently the Council of the Society picked the right man for the work when they asked Colonel Moores to undertake it. The result is creditable to all parties.

Asepsis and How to Secure it. By H. W. Carson, F.R.C.S. (London: The Scientific Press, Ltd. 1914. Pp. 56. Price 1s. net.)

So many changes have been introduced into the daily ritual of the operating theatre during the last few years that an account of present-day practice in a concise form is most acceptable to all who wish to be and to keep abreast of what is best in this sort of work. Mr. Carson is a guide whose qualifications for the task he undertakes are of the best, and his booklet is well worthy of his high reputation. His gospel is simple, straightforward, and sufficient; surgical salvation, as at present conceived of, is to be attained by accepting and following it, though the future may, of course, produce something even nearer to perfection. His diagram of a theatre unit is not very good: there is wasted space, no surgeon's dressing room is shown, and the anæsthetising room is of an awkward shape and has neither cupboard nor sink. Apart from this, the book practically defies criticism, and should prove of the greatest benefit to all nurses who are doing, or may some day do, "theatre work."

The Principles and Practice of Medical Hydrology. By R. Fortescue Fox, M.D. (London: University of London Press. 1913. Pp. 295. Price 6s. net.)

As has been on several occasions noted in the columns of The Hospital, there has been a distinct revival during the last few years in the science and practice of balneology. Much more attention is being paid to the rational principles which underlie, or ought to underlie, the study of this subject; and several monographs on British and foreign spas have made their appearance. Then, too, the discovery of radio-activity in the waters at Bath, and the evident determination of the authorities there to exploit it, have drawn attention to the national asset which the many mineral springs of Britain ought to be, and to their comparative neglect, in favour of foreign resorts of no greater, and even smaller, potency.

The scientific study of medical hydrology has really hardly begun; when it is seriously taken in hand there is little reason to doubt that progress as great will be made as that which other branches of medicine have achieved in recent years. The use of vague, grandiloquent, but really meaningless, phrases seems firmly rooted among balneologists, who would do better to admit their inability (as yet) to explain the exact *rationale* of many undoubted therapeutic actions of various baths than to resort to language perilously close to nonsense. No better illustration of this tendency can be given than a passage from the writings of Winternitz, quoted by Dr. Fox. He says: "Thermal stimulations are, therefore, indicated when innervation requires to be strengthened, suspended, or altered." No wonder the surgeon and the bacteriologist laugh outright when they see such rubbish written by physicians. Nevertheless, on the whole, Dr. Fox steers fairly clear of this misuse of words and corresponding confusion of thought; but he does not succeed in avoiding it altogether. The greater part of his book, however, consists of practical information about the various spas of Britain and elsewhere, with useful comments as to the types of cases which will get benefit from each. This is what makes his book really valuable and thoroughly justifies him in publishing it.

The Debenture: Its Use and Abuse. By H. W. Jordan. (Jordan and Sons.)

The expressive American phrase "sucker" is applied to anyone whose knowledge of, and capacity for, business are insufficient to protect his interests or property from fraud. The medical profession, both in America and here, is generally supposed to share with the clergy the distinction of containing the greatest percentage of "suckers." Whether this is true or not, there are points in debenture law and practice which might trip even the most astute financier; and the simplification of the whole subject in this modest pamphlet is admirable.

### The Personal Factors in Institutional Life.

Alice Ottley: A Memoir. Compiled by Mary E. James. (London: Longmans, Green and Co. 6s.)

The personal element in institutional life forms the subject of this volume, and, indeed, it rarely happens that a biography of a woman combines so many absorbing features as does this memoir. Miss Ottley not merely left her mark on the world by reason of her long association with the Worcester High School for Girls, extending as it did over a period of thirty years, during which time the school was under her charge as head mistress, but she made her great influence for good felt in countless other directions. Miss Ottley's life affords a truly remarkable example of the highest and most ennobling type of personal service, and the memoir should be read by all who are capable of learning and appreciating the untold good which can be wrought by a simple-minded worker of energy, character, and ideals. One of the most valuable features of the book, in our judgment, is the appendix, which contains a selection of letters written by Miss Ottley to former pupils and others. These letters contain much advice and suggestion on a variety of subjects, and will be studied with admiration and gratitude by many who may never have been privileged to come within the sphere of influence of the saintly subject of this book. The appendix has, in fact, the practical value of being a series of examples of the way in which character and common sense can be applied to the hundred and one questions which are presented to the administrative heads of institutions who have established personal claim to regard in the minds of those under their control.

